# Using the *talpid^2 ^*as novel model for determining the cellular and molecular etiology of Oral-facial-digital syndrome

**DOI:** 10.1186/2046-2530-4-S1-P41

**Published:** 2015-07-13

**Authors:** EN Schock, CF Chang, JN Struve, J Chang, SA Brugmann

**Affiliations:** 1 Developmental Biology, Cincinnati Children's Hospital Medical Center, Cincinnati, OH, USA; 2 Surgery, Cincinnati Children's Hospital Medical Center, Cincinnati, OH, USA; 3 University of Cincinnati, Cincinnati, OH, USA

## Objective

Oral-facial-digital syndrome (OFD) is a ciliopathy characterized by craniofacial abnormalities including cleft lip/palate, glossal defects, and absent/dysmorphic or supernumerary teeth. In addition, these patients have several other abnormalities typical of a ciliopathy including polysyndactyly, hypoplasia of the cerebellar vermis (molar tooth sign), cardiac defects and polycystic kidneys. Recently a subset of OFD cases have been linked to mutations in the centriolar protein, calcium C2-dependent domain containing 3 (C2CD3). Interestingly, our previous work identified a mutation in C2CD3 as the causal genetic lesion for the avian *talpid^2 ^*mutant. Based on this common genetic etiology, we re-examined the *talpid^2 ^*mutant for OFD-like phenotypes. We found that almost all phenotypes are conserved between *talpid^2 ^*embryos and OFD patients. In light of this finding we utilized the *talpid^2 ^*to examine the cellular basis for the craniofacial phenotypes present in OFD.

## Methods

Using both *in vivo *and *in vitro *methods we analyzed specification, migration, proliferation and differentiation of cranial neural crest cells (CNCC) when C2CD3-dependent ciliogenesis was impacted.

## Results

Our studies suggest that whereas disruptions of C2CD3-dependent ciliogenesis did not affect CNCC specification or proliferation, it did affect CNCC migration and differentiation. Migrating *talpid^2 ^*CNCCs were more disperse than control CNCCs and their migration was impaired. Furthermore, *talpid^2 ^*CNCC derived cartilages are larger relative to controls.

## Conclusions

Taken together, these findings suggest that the avian *talpid^2 ^*mutant is a bona fide, novel model for OFD and that aberrant CNCC migration and differentiation could contribute to the pathology of C2CD3-dependent human OFD.

